# A qualitative study of the multi-level influences on oral hygiene practices for young children in an Early Head Start program

**DOI:** 10.1186/s12903-019-0857-7

**Published:** 2019-07-26

**Authors:** Tracy L. Finlayson, MarkJason Cabudol, Jenny X. Liu, Jeremiah R. Garza, Stuart A. Gansky, Francisco Ramos-Gomez

**Affiliations:** 1 School of Public Health, Health Management and Policy, San Diego State University, 5500 Campanile Drive, San Diego, CA 92182-4162 USA; 2 University of Washington (UCLA School of Dentistry at the time of this work), Seattle, WA USA; 3 Department of Social and Behavioral Sciences, School of Nursing, Institute for Health & Aging, University of California San Francisco, San Francisco, CA 94143 USA; 4 Department of Health Policy and Management, UCLA Fielding School of Public Health, University of California, 650 South Charles E. Young Drive South, Box 951772, Los Angeles, CA 90095-1772 USA; 5 School of Dentistry, University of California San Francisco, Box #1361, San Francisco, CA 94143 USA; 6 Section of Pediatric Dentistry, University of California Los Angeles, School of Dentistry, 10833 Le Conte Avenue, Box 951668, CHS Room 23-020B, Los Angeles, CA 90095-1668 USA; 7 Center to Address Disparities in Children’s Oral Health (known as CAN DO), University of California San Francisco, School of Dentistry, Box #1361, San Francisco, CA 94143 USA; 8 Philip R. Lee Institute for Health Policy Studies, University of California San Francisco, Box #0936, San Francisco, CA 94143 USA

**Keywords:** Oral hygiene, Qualitative, Early head start, Preschool-age children, Mothers, Home visitors

## Abstract

**Background:**

Individual child-level risk factors for Early Childhood Caries (ECC) have been studied, but broader family- and community-level influences on child oral hygiene behaviors are less well understood. This study explored multiple levels of influence on oral hygiene behaviors for young children in Early Head Start (EHS) to inform a future behavioral intervention targeting children from low-income families.

**Methods:**

Twenty-four semi-structured interviews were conducted with mothers of children under 4 years old, enrolled in the home visitor (HV) component of one EHS program in Los Angeles, CA, who participated in the BEhavioral EConomics for Oral health iNnovation pilot study (BEECON) in 2016–7. Audio-recordings of interviews were translated if needed, and transcribed in English, and coding and analysis was facilitated by Dedoose qualitative software. This investigation used general thematic analysis guided by the Fisher-Owens child oral health conceptual framework to identify influences on oral hygiene behaviors for the young children.

**Results:**

Many mothers reported brushing their children’s teeth twice/day, and concern that most children frequently resisted brushing. They identified children being sick or tired/asleep after outings as times when brushing was skipped. Several child-, family-, and community-level themes were identified as influences on child oral hygiene behaviors. At the child-level, the child’s developmental stage and desire for independence was perceived as a negative influence. Family-level influences included the mother’s own oral hygiene behaviors, other family role models, the mother’s knowledge and attitudes about child oral health, and mothers’ coping skills and strategies for overcoming challenges with brushing her child’s teeth. Overall, mothers in the EHS-HV program were highly knowledgeable about ECC risk factors, including the roles of bacteria and sugar consumption, which motivated regular hygiene behavior. At the community-level, mothers discussed opportunities to connect with other EHS-HV families during parent meetings and playgroups that HV coordinated. A few mothers noted that EHS-HV playgroups included brushing children’s teeth after snacking, which can be a potential positive influence on children’s hygiene practices.

**Conclusion:**

Child-, family- and community-level factors are important to consider to inform the development of tailored oral health preventive care programs for families in EHS-HV programs.

**Electronic supplementary material:**

The online version of this article (10.1186/s12903-019-0857-7) contains supplementary material, which is available to authorized users.

## Background

Early Childhood Caries (ECC) is a multi-factorial disease, and remains a prevalent health problem in the United States (US) that disproportionately affects young children in lower income families and those from racial/ethnic minorities [1–3]. According to the American Academy of Pediatric Dentistry (AAPD), ECC is defined as “the presence of one or more decayed (noncavitated or cavitated lesions), missing (due to caries), or filled tooth surfaces in any primary tooth in a child 71 months of age or younger.” [4] The Fisher-Owens and colleagues’ conceptual framework identified five health determinant domains—genetic and biological factors, social environment, physical environment, medical and dental care system, and health behaviors [5]—that can influence a child’s oral health status over time, operating at child-, family-, and community-levels (see model Figure at: https://pediatrics.aappublications.org/content/pediatrics/120/3/e510/F1.large.jpg?width=800&height=600&carousel=1). While several domains identified in the Fisher-Owens and colleagues’ framework are either non-modifiable (e.g., genetics/biology) or more challenging to change without systematic efforts or policy interventions (e.g., social and physical environments, the healthcare system), health-influencing behaviors can readily be modified to improve oral health status. Behavior change has been an intervention focal point for ECC prevention among children in the last two decades, but results have varied [6, 7]. Developing effective early preventive programs for children in high-risk families remains a priority.

For infants and young children, parents primarily drive oral hygiene routines. Mothers are often primary caregivers, playing a key role in cleaning infants’ mouths and brushing children’s teeth [8–10]. While the AAPD recommends starting to clean an infant’s teeth as soon as they erupt [11], this is not common knowledge or practice in many families [12]. Even when parents report knowing they should clean a young child’s mouth, gums, and teeth, and know ECC risk factors, cleaning and brushing regularly remains challenging, with parents identifying many barriers relating to the family environment [13, 14].

Poor oral hygiene behavior is an established risk factor for developing ECC [15]. Educating parents on proper hygiene techniques, including daily brushing, is necessary for promoting children’s oral health starting in early childhood and preventing ECC from developing into cavities later in life [16, 17]. Previous research has focused on individual child-level ECC risk factors, but broader family- and community-level influences on ECC and young children’s oral hygiene behaviors are less well understood [9]. Castilho and colleagues recently reviewed family-level influences on child oral health, finding parents’ dental beliefs and habits affected their children, and interventions should address the entire family and home context [18]. Notably, nationwide, many infants and toddlers spend substantial time outside their homes, in preschools, or with relatives, being cared for by non-parental caregivers [19]. Preschool programs which are well-positioned to influence child and family oral health practices should be explored; this is particularly true for programs that also deliver curriculum beyond the classroom at children’s homes, working closely with parents and families in their own environment to promote child health.

Early Head Start (EHS) is a federally-regulated US public program providing comprehensive social, educational, and health services for low-income pregnant women and children age three or younger [20]. Federal oral health performance standards promote preventing and treating children’s oral health problems [21, 22]. EHS program services can be delivered as home-based care, center-based care, family childcare (licensed providers offer care in their homes), or a combination. Home-based EHS programs, which represent 36% of all US, and 50% of California, EHS-funded enrollment slots employ Home Visitors (HVs). A dedicated HV visits weekly [23] for 90-min and leads two group socialization activities per month for families. HVs incorporate oral health strategies and activities into their lesson plans, which include promoting early and regular oral health examinations at a dental home (regular provider of comprehensive oral health care [24]), toothbrushing with fluoridated toothpaste according to age-specific recommendations, and educating parents about their children’s oral health [25–28]. Thus, EHS programs present an important opportunity to identify, target, and efficiently reach parents of young children at high ECC risk.

Prior EHS program research documented mandated programmatic activities to promote oral health are not always optimally implemented. Kranz and colleagues found low participation in oral health activities in North Carolina EHS programs [29]. They identified EHS program staff factors like high knowledge and efficacy as positively associated with more frequent oral health-promoting activities [30]. Mofidi and colleagues [31] noted challenges in EHS staff and parent communication around oral health, and confusion around oral health standards. Head Start and EHS health program managers report oral health promotion is a common health service component, and 75–90% of HVs taught proper toothbrushing technique to families [32]. Program managers reported basic hygiene instruction was provided to all families, but more oral health trainings and services were provided if a program viewed ECC as a significant problem [32]. Other research has shown that brief trainings (one-time, three-hour session) for EHS HVs can effectively significantly improve HVs’ oral health knowledge [33], though it is not clear how that translates to practice. While EHS program characteristics have been studied, less is known about the specific contexts for children participating in EHS HV programs and the myriad factors that influence their hygiene behaviors and ECC risk.

The Children’s Partnership recently called for expanded support for oral health components promotion in HV programs serving young children, including in EHS [34]. EHS performance standards require family engagement in the health service components, including oral health [35]. A major EHS home-based program benefit is the HVs’ ability to connect directly with families in their homes. While HVs can offer health information, instruction, and support, families still have to make the behavioral changes. Thus, finding additional ways to motivate and encourage sustained behavior change in these families are urgently needed.

This study took place at an EHS program that offered home-based services located at a multi-site Federally Qualified Health Center (FQHC) in Los Angeles, California. As of 2017, this site provides comprehensive medical care to 26,000 children and adults. Here, we focus on families in the EHS HV program and examine the multi-level influences on parents’ hygiene behaviors for their children. The aim of this study was to better understand how and where to intervene and support optimal oral hygiene practices, defined as twice daily toothbrushing with an age-appropriate amount of fluoridated toothpaste, using a qualitative design. This methodology is ideal at the formative phase for exploring a range of factors at different levels. Following the Fisher-Owens and colleagues’ conceptual framework, this paper explores child-, family- and community-level influences on oral hygiene practices for young children participating in an EHS HV program.

## Methods

### Study design

We conducted a qualitative study using semi-structured interviews with parents of children under 4 years old in one EHS in Los Angeles, CA. This study investigated child-, family-, and community-level influences on the EHS child’s oral hygiene practices. The in-depth interviews were part of formative research conducted to inform the design of an intervention aimed at improving parental behaviors for oral hygiene for their young children. The academic institution’s Institutional Review Board approved this study.

### Participant recruitment

Fourteen EHS HVs verbally advertised the study and disseminated a project flyer to each family. Using this non-probabilistic purposive sampling approach, 122 families returned a form to their HV authorizing the research staff to contact them. Two trained research assistants (RAs) contacted each parent and screened them for eligibility; criteria were: parent must be child’s primary caregiver; must be at least 6 months old but less than 4 years old (if more than one child was in this age range, the oldest child was eligible); and their child must be enrolled in or waitlisted for the EHS HV program.

Of the 122 families interested in participating, 74 caregivers (all of whom were mothers), gave verbal consent, and completed a computer-assisted telephone interview (CATI) including socio-demographic questions (61% response rate). At the end of the CATI, respondents were then invited to participate in an in-person interview. Of the 42 expressing interest, in-person interviews were conducted with a convenience sample on a rolling basis between November 2016 and February 2017 until the predetermined 25 interviews was reached to gain adequate data saturation and breadth and depth of input on desired topics.

A preliminary semi-structured interview guide was initially developed in English, translated to Spanish by a professional translator, and back-translated to English to ensure accuracy. The guide was iteratively pre-tested, with both English- and Spanish-speakers, to assess length, respondent comprehension of question phrasing and probes, relevance, and question flow. The final interview guide (Table 1) changes were translated into Spanish by one bilingual staff member, and reviewed independently by two other bilingual staff. Many topics were included to understand the family composition, engagement with EHS staff and other EHS families, parents’ oral health knowledge and practices for themselves and the EHS child, the child’s daily toothbrushing routines, brushing challenges and strategies, and preferences about different incentives. Two trained RAs conducted in-person interviews (*n* = 21 in Spanish, *n* = 4 in English) in a private room in the FQHC. Written informed consent was obtained from each participant. Both RAs facilitated each interview: the bilingual English/Spanish female RA led the questioning for all interviews, while the other RA prepared all study materials, audio-recorded the interview, took field notes, and if necessary, engaged with the participants’ child(ren) to reduce interview distractions. Interviews lasted approximately 1 h (mean = 51 min, range: 27–84 min), including an option for a short refreshment break. Participants received a $40 grocery store gift card as compensation for their time. An experienced translation service translated audio recordings to English if necessary, and transcribed the recordings.

**Table 1:** Select questions from semi-structured interview guide

Sample Demographics	
1. Can you please tell me about your family or the people you live with?	
◦ Who do you live with?	
◦ How many children do you have?	
◦ How old is your child in the EHS program?	
Early Head Start Parent-to-Parent Communication	
2. How well do you know some of the other parents and children in the Early Head Start program?	
◦ How often do you see them? Talk to them?	
◦ What kinds of things do you talk about with them?	
Family Oral Health Practices	
3. As you know, our research is trying to learn more about oral health. Can you tell me about what you do regularly to maintain your oral health?	
◦ When do you usually prefer to brush? Why?	
◦ How similar is it for the other adults in your family?	
◦ Would you make any changes to maintaining your oral health?	
4. Our study is focused on your children under age 4. How do you or your family maintain healthy teeth for your child/your children under 4?	
◦ What times of the day does brushing usually happen (morning, evening, both, other times?)	
◦ Why are those the best times to brush?	
◦ Why do they brush that often?	
5. How did your child learn how to brush his/her teeth?	
◦ At what age did you start brushing his/her teeth? Why?	
◦ At what age did or do you expect a child to start brushing his/her own teeth? Why?	
6. What do you think are the most important aspects of caring for your child’s teeth? Why?	
◦ What are your main concerns?	
◦ How likely do you think that your child will have any challenges with their teeth? Why?	
7. You mentioned that you have older children. Are there experiences that you’ve had with their toothbrushing habits that have influenced what you now do with your younger child? IF YES: Can you tell me about that?	
Brushing Narratives	
8. To help me understand more about parents’ lives, I’d like to know more about your daily routine. Please think about this morning. Could you walk me through what went on as you were getting ready for the day?	
◦ Tell me about the children. Who got the young children ready and how did that go?	
◦ Where did toothbrushing fit into your daily morning routine?	
▪ Who takes care of your child/children’s toothbrushing?	
◦ Did the toothbrushing actually occur as planned? What happened?	
▪ How easy or difficult was it to get the brushing done?	
▪ What did you do to keep him/her engaged while brushing? (e.g., Did you do another activity while toothbrushing?) How well did that work?	
▪ How long did this all take?	
◦ Is this what morning usually look like? Why or why not?	
▪ How similar or different is it on the weekdays vs. the weekends?	
9. Similarly, I want to get a general idea of what happens in the evenings. Please think back to last night before bed. Could you walk me through what went on as you were getting ready for bed?	
◦ Tell me about the children. Who got the young children ready for bed and how did that go?	
◦ Where does toothbrushing fit into your nightly routine?	
▪ Who takes care of your young child/children’s toothbrushing?	
◦ Did the toothbrushing actually occur as planned? What happened?	
▪ How easy or difficult was it to get the brushing done?	
▪ What did you do to keep him/her engaged while brushing? (e.g., Did you do another activity while toothbrushing?) How well did that work?	
▪ How long did all this take?	
◦ Is this what nights usually look like? Why or why not?	
▪ How similar or different is it on the weekdays vs. the weekends?	
10. What usually gets in the way of brushing your young child’s teeth?	
11. What ways can you overcome these challenges?	

See Additional file 1 for Spanish version

### Data analysis

Qualitative analysis followed transcription and translation of the interviews. We used Dedoose (SocioCultural Research Consultants, Manhattan Beach, CA, USA) mixed methods software for data management and to conduct all analysis of textual data. One RA with qualitative training and experience using Dedoose iteratively reviewed all transcriptions for interview context and recorded analytic memos to note and further discuss impressions and ideas relating to the conceptual framework with the research team in the early data interpretation stage. Codebook development was based on recorded notes from iterative transcription reviews and the interview guide. Codes were attached to excerpts using general thematic analysis, grouped into preliminary themes, and discussed with the research team to reach theme consensus. Our thematic analysis was guided by the Fisher-Owens and colleagues’ conceptual framework [4], selected because it is a well-established multi-level model that captures many oral health determinants. A team member with experience and expertise in qualitative research methods and oral health also reviewed all transcripts, codes, and final thematic interpretation. Exemplary quotes were extracted to illustrate key themes.

Demographic data from telephone surveys were descriptively summarized in Qualtrics (Qualtrics, Provo, UT). Three phone surveys which were incomplete due to phones disconnecting or participants declining to answer, were excluded from the sociodemographic analyses (*n* = 22).

## Results

This study took place at a predominantly (86%) home-based EHS program (11% family child care and 3% prenatal women services). Sociodemographic characteristics of in-depth interview participants were summarized in Table 2. Almost two-thirds (64%) of mothers were between ages 30–39; nearly all (91%) were born outside the US. About one-third (36%) completed high school, 41% declined to report family annual income (though they met EHS eligibility income levels), while 36% had four people living in their household and 52% had five or more. About half (54%) of mothers reported that their oldest child in EHS was 24 to 35 months years old. One child was waitlisted for the EHS HV program at the time of interview; the remainder were currently enrolled. Two children had Down’s Syndrome.

**Table 2:** Participant characteristics (*N* = 22)^a^

	n	%^b^
Interview language^c^		
Spanish	18	82
English	4	18
Maternal age^d^		
20–29	5	23
30–39	14	64
40–49	3	14
Education^d^		
Less than high school	8	36
Completed high school	8	36
Some college, but no degree	4	18
Associate degree	1	5
Bachelor degree	1	5
Birth Country^d^		
United States	2	9
Other Country	20	91
Family annual income^d^		
Less than $10,000/year	1	5
$10,001–$20,000/year	8	36
$20,001–$30,000/year	4	18
Refused	9	41
Household Size^c^		
3	3	12
4	9	36
5	6	24
6	3	12
7	2	8
8	1	4
9	1	4
Oldest child age in Early Head Start^d^		
0–11 months	2	9
12–23 months	7	32
24–35 months	12	55
36–47 months	1	5

^a^ 3 surveys were missing

^b^ Percentages may not add to 100 due to rounding

^c^ Collected via transcriptions

^d^ Collected via telephone surveys

**Table 3:** Summary of themes identified that influence children’s oral hygiene behaviors, by level, from Fisher-Owens et al. framework

Level	Influences on health behavior	Themes
Child	Health behavior	Child’s usual oral hygiene routine
Child	Stage of development	1. Desire for independence
Family	Family function and roles	2. Mothers’ role 3. Other family role models
Family	Beliefs and practices	4. Oral health knowledge 5. Oral health attitudes
Family	Coping skills	6. Coping skills and strategies
Community	Community Oral Health Environment	7. Community Oral Health Environment and Early Head Start Home Visitor program norms
	Social Environment	8. Social Environment
	Health system characteristics	9. Health system characteristics

Mothers’ reports of their EHS children’s oral hygiene routines were presented, as well as factors that affected those behavioral routines. The seven themes identified were presented, organized by level of influence (child-, family-, and community-levels), supported by select illustrative quotes. Themes are summarized in Table 3.

### Children’s oral hygiene routines

Mothers’ narratives included descriptions of their children’s oral health practices. Many mothers shared that they started cleaning their children’s mouths and teeth before they turned one, mainly using a finger toothbrush or cloth, then switched to brushing around age one. Most mothers indicated that they were usually the main person who brushed the child’s teeth, and that the child’s oral hygiene routine commonly included brushing in the morning and before bed at night (i.e., twice per day). Several talked about an additional brushing episode after every meal when at home if possible, and particularly preferred brushing in the afternoon. Some delayed brushing times to later in the mornings on weekends, when activities were less routine.

Interviewer: *Why is the afternoon better for you?*

Participant: *It’s because everyone’s home. It’s when everybody has already eaten and I can take the time to check those things.* -Mother of less than 1 year old child

*I feel like the weekdays are easier because we’re following a routine. We’re thinking, “Ok, we have to brush our teeth, read, and go to sleep.” On the weekends, we’re more relaxed. My brother or a cousin will come to visit or we’d go to a party somewhere. There’s less control on the weekends.* – Mother of less than 3 year old child

Mothers also discussed deviations from their child’s regular hygiene routine. A few mothers mentioned child sickness to be a brushing barrier because children would not be interested in doing anything (i.e., brushing, eating, sleeping) following their usual routines.

*It’s only when she’s sick. That’s when she doesn’t want to brush her teeth. She doesn’t want anything. Even just opening her mouth when she’s sick becomes very difficult. She doesn’t want to eat or sleep. She doesn’t want to brush her teeth. She doesn’t want anything when she’s sick.* - Mother of 2 year old child

Brushing was also more problematic for mothers when children were exhausted and/or asleep, and oftentimes would be skipped under these circumstances. Mothers did not want to wake their sleeping children to brush.

*When you suddenly go out or you just have to go out, the time to do it [opportunity to brush] passes because he’s already asleep and he won’t get up until the next day.*

- Mother of 2 year old child

### Child-level influences

Mothers identified child-level influences on hygiene routines related to the child’s stage of development and overall level of cooperation. Most mothers indicated that brushing was often a challenge due to their child’s resistance and increasing sense of independence.

### Stage of development

Several mothers mentioned the child’s stage of development and their growing desire for independence as a barrier to brushing. Child resistance was another major brushing barrier identified by many mothers. Older children were more verbal, while younger children would throw tantrums and many would keep their mouths shut.

*He has episodes of when he doesn’t get it his way; it’s either his way or the highway. An example would be maybe a couple of days ago he was like,"* *No!" I told him,"* [NAME]*, I’m going to wash your teeth." He goes,"* *No! I want to do it," and I go,"* *No, I want to do it." I got persistent with him the same way he got persistent with me so he was just like,"* *Fine! No!" and he walked right out of the room.* - Mother of 2 year old child

### Family-level influences

Family-level influences on young children’s oral hygiene behaviors included the role of family members and the presence of role models for brushing, the mother’s oral health-related beliefs and own hygiene practices, and the mother’s coping skills for overcoming barriers to brushing her child’s teeth.

### Mother’s role

Mothers expressed that they were primarily responsible for their child’s oral hygiene, and many noted that they felt they were important positive role models for their young children’s oral hygiene. Mothers would often model brushing their own teeth.

*Watching us because if I go to the restroom, then he goes to the restroom. If I go to the kitchen, then he goes to the kitchen. Every time I go brush my teeth, he’s there asking for my toothbrush. I say, “It’s not your turn! Let me do mine!” He watch[es] me and he opens his mouth. He’s making noises and that’s what he learned.* – Mother of 1 year old child

### Family role models

A few mothers discussed how their older children’s brushing experiences helped to shape their younger children’s oral hygiene routine. Older children served as positive role models and would help reinforce brushing as a habit for their younger siblings.

*I think yeah the habits that have been influenced is that with her [older sibling] - she has been really good in listening to directions. She’s also very organized so she follows, like,"* *Okay, the next thing we have to do is brush our teeth." So I think the habits I have done with her, the good habits and the discipline I think have helped him [focal child]. He sees so he is like"* *Oh she is going to go brush her teeth, so now I have to go." So I think that has really helped me in the way that I don’t really have to tell him,"* *Oh, go brush your teeth." Like he knows,"* *Oh I’ll follow my sister." Or, um, he’ll learn faster from seeing her.* – Mother of 2 year old child

Poor dental experiences with older children also motivated mothers to improve dental hygiene regimens for younger siblings, increased maternal awareness about oral health more generally, prompted them to start oral hygiene routines at earlier ages with the younger siblings.

*For instance, with the big one [older sibling] it happened to me that I didn’t brush her teeth with a toothbrush, but with a little cloth when she had a few teeth. For me, it wasn’t important to brush her teeth, because I thought, ‘She’s still little’ and I would only do it with the little cloth every day. And it was hard for me to find the time to brush her teeth, until I saw the little black spots, like the early stages of cavities. And I thought, ‘Oh.’ So I am more aware of this one, because I know this can happen.*

– Mother of less than 1 year old child

A few mothers mentioned their husbands were barriers to maintaining their child’s daily toothbrushing because of how relaxed they were about it.

Participant: *He [child] has like a routine with me. That’s the word that I was looking for. With his dad he’s more laidback. With his dad he’ll do whatever he wants. I keep him more on a schedule and on a routine with me.*

Interviewer: *How do you feel about that?*

Participant*: I feel that his dad has to be on the same page as me because I feel like – he knows it and then they get used to it because he knows when his dad is around and I’m home too he knows if he wants to do whatever he wants. He’ll start crying and he’ll go to his dad because he knows his dad is like,"* *Just do whatever you want. I’ll watch you."*

– Mother of 1 year old child

*In the mornings daddy has that responsibility. Daddy is a bit more laid back… He is more like, how do I say it. For him he’s more like, “They’re just kids. They’ll eventually learn. If we didn’t do it this morning, it’s okay, we’ll get tomorrow, you know? Or we’ll do it later.”* – Mother of 2 year old child

Not all fathers were relaxed, however, and some mothers indicated that fathers played a consistent role and was in charge of brushing the child’s teeth as part of their normal daily routine.

### Knowledge of child oral health

Caries prevention was a primary motivating factor for brushing young children’s teeth. Mothers in the EHS HV program were quite knowledgeable about ECC risk factors, including the role of bacteria and sugar consumption. Many mothers believed morning bad breath resulted from the accumulation of bacteria from the previous night’s sleep and preferred their children to start each day with a clean mouth. Many described the role of bacteria and microbes and plaque specifically, including bacteria transmission in saliva, as part of the caries development process.

[explaining why she brushes child’s teeth twice per day] *Well, because at the beginning the pediatrician told me, as did the dentist, that when she wakes up she has all the microbes that her saliva produces throughout the night. And at night, after she has her last thing to eat, I have to do it and I shouldn’t give her anything. They said even if she drinks milk, some of it will be left in her teeth. That’s why they say that at night after I brush my teeth I shouldn’t give her anything at all, except for water if she wants. So that nothing stays there, like milk.* –Mother of less than 2 year old child

Mothers were motivated to brush their children’s teeth at night because they had eaten food all day and wanted their children to go to sleep with a clean mouth. Mothers understood that consuming excess candy and sugary beverages can cause cavities. They also said that they maintained their children’s oral health by limiting their sugar intake, though some mothers struggled with managing this behavior.

*Well, we try to keep them healthy by making sure that she doesn’t eat too much candy. In the evenings she doesn’t drink juice nor eat any food after she brushes her teeth. We don’t give her anything except for milk, if at all.* – Mother of 1 year old child

*They* [children] *don’t drink juice and only with water when they want to. I don’t give them many sweets because I know that causes cavities. They don’t drink sodas. I try to give them the healthiest* [foods] *that I can.* – Mother, child age unavailable

### Attitudes about child’s oral health

For many mothers, their main concerns for their child’s oral health were related to preventing cavities and poor oral health. A few mothers discussed that their child’s oral health was important because oral health was connected to overall health, and that poor oral health could lead to future general health problems.

*Honestly, when my daughter had a few caries* [sic]*, and I was coming every month so they could check on her, I was very concerned. I thought, “She’s so little, and I don’t want her to suffer with caries.” I always did what they told me to.* – Mother of 2 year old child

Participant: *Also her own* [overall] *health. If her dental health is fine, then everything else is going to be ok. If your dental health is bad, she can get cavities which can bring her serious health problems.*

*Interviewer: Oh, ok. Why do you think that there is this connection?*

Participant: *This connection?*

Interviewer: *Between your general and oral health.*

Participant: *Oh, ok. I think so because that’s what doctors say, right? If you have bad dental health, it can bring infections and it can- Everything is connected. It’s one body, isn’t it?* – Mother of 1 year old child (with Down’s Syndrome)

These attitudes about the importance of oral health motivated regular oral hygiene behaviors for their young children.

Mother’s expectations for when her child can brush his/her own teeth was another important belief that influenced child oral health hygiene practices. Mothers varied widely in their beliefs about the appropriate age of independent brushing among children. Most parents expected children to brush independently at 5 years old, but some mothers didn’t know there was any recommended age or the “right” age, and answers ranged from one to 5 years of age.

### Coping skills/strategies

Mothers discussed multiple strategies and distraction techniques to complete brushing their children’s teeth. Some mothers engaged their children during morning toothbrushing through storytelling. Storytelling was an effective strategy for some because it distracted the child, allowing the parent to carry on with the brushing, and kept the child’s attention after brushing. Storytelling involved using both positive and negative frames to describe planned activities or consequences of not maintaining good hygiene habits.

*Well, one day she didn’t want to* [brush teeth]*. At the beginning, she didn’t want to because the toothpaste was different. She said, “It stings, it stings.” She didn’t want to so I told her, “Do you know what we’re going to do tomorrow?” So I said this or that and then I noticed that she stayed still. I did it the next day and realized that it was working. That’s why I do it.* – Mother of 2 year old child

*I tell her a story or I play a song* [on mother’s phone] *for her, something like that. But in the mornings when we’re rushing, I try to tell her something, “Oh my, did you know that this or that happened to such and such girl. She didn’t take care of her teeth so now she’s in pain.” And she’s just like, “Ahh. And did she bleed?” I said, “No, she didn’t bleed but she is in pain and she’s going to have to go to the dentist because she doesn’t know what’s wrong.” And even after we finish, she still wants me to continue telling her about it! But I do try to come up with something.* – Mother of 3 year old child

In addition, mothers engaged their child by singing and allowing their child to watch videos while brushing. Several mothers showed their child photos on their phone or turned on the television. Some showed dolls’ teeth as an example of good oral health vis-à-vis the aesthetic of white beautiful teeth, which was contrasted against poor oral health hygiene outcomes that could result in teeth that look “yellow” or “ugly.” One mother mentioned to her daughter that she planned to brush her daughter’s teeth first so that her daughter could then brush her doll’s teeth.

*He* [target child’ sibling] *saw a video that it’s good to sing when you’re, not really sing, but like a song when you brushing your teeth. That’s a video that we saw for kids so they don’t get bored to do it for like 3 min.* -Mother of 1-year-old child

*Well, sometimes I tell her that- I use her dolls and I tell her,* “*Look, your dolls have very beautiful teeth,” I say to her, “That’s because your dolls brush their teeth and if you don’t brush [your teeth], your teeth are going to look very ugly, they’ll get all yellow and will look bad.” She says, “Mommy, I don’t want that. I don’t want my teeth to look ugly.” “Well, you have to brush them” I say to her. And then she goes to the bathroom and I tell her, “Let’s go brush your teeth.”* - Mother of 2-year-old child

Nearly all mothers described these strategies as tools for distracting their children long enough for them to brush their teeth. Some mothers, however, lacked effective solutions to overcome brushing barriers, leading to abandoning brushing and waiting until the child was cooperative to try brushing again.

### Community-level influences

At the community-level, potential influences were identified related to community oral health environment and social environment within the context of the EHS HV program, and health system characteristics. Additionally, culture plays a role at the community-level of influence as well as at the individual-level. Cultural beliefs, shared in communities, can influence health beliefs and practices.

### Community oral health environment

A few mothers described how EHS HV provided oral hygiene demonstrations during the home visit, promoting good oral health in the home with the child and family.

*They* [children] *didn’t want to brush their teeth, but a worker* [Home Visitor]*, she visited us often, she brought a dinosaur with big teeth and a big toothbrush. She put shaving gel instead of toothpaste on the toothbrush so she showed them, ‘Oh, the dinosaur washes their teeth.’ She colored in the dinosaur’s teeth with a crayon, ‘You see that’s the food and you get dirty on the teeth so we’re going to wash it off.’ So something like that and they go, ‘Oh the dinosaur is washing their teeth so I got to wash my teeth.’* – Mother of 2 year old

Mothers noted there were opportunities to interact with other EHS families in the HV program, and for EHS HVs to provide health and child development information. Mothers mentioned mostly seeing other EHS parents and children at playgroup sessions and parent meetings, though many did not interact regularly with other EHS families otherwise. HVs organized playgroups with educational activities for parents and children on a regular basis (weekly or twice per month), and as an opportunity for parents to socialize with other parents.

The monthly parent committee meetings offered information about various topics, such as immigration, housing, basic human rights, and childcare, though those were less well attended than the playgroups.

*Sometimes, when you go to the* [parent committee] *meetings that they do they occasionally provide help or assistance. For instance, now, with immigration, people come to talk about our rights or there are people who come to talk about leasing controls. Also, there are people who come to talk to us about children, like how to detect when they are suffering from autism. So there are different classes that they give us so that we can learn things that one doesn’t know.* – Mother of less than 1 year old child

Many mothers discussed sharing concerns about their child and sought advice from other mothers at playgroups or at parent meetings, and from EHS home visitors. No mothers mentioned discussing oral health or oral hygiene information in these groups, but did discuss other child health concerns and developmental milestones. Some identified that the HV provided the same health information and recommendations as their healthcare providers, suggesting that HVs were seen as providing reliable and provided some helpful child health education and support.

A few mothers noted that oral hygiene was specifically discussed as part of the EHS playgroup routine. A couple mothers indicated that playgroups incorporated brushing and cleaning the children’s mouth after snack as part of the routine. The playgroup offered a supportive environment outside the home as a positive influence on promoting regular oral hygiene practice.

Participant: *Usually we’ll go to – the little playgroup usually happens on* [location]*? We’ll get there and the kids will play and read. They’ll have their little snack and then after the snack they’ll go and play again. They’ll sing a song to them and that’ll be it.*

Interviewer: *Wow. That’s a lot of activities.*

Participant: *Yeah.*

Interviewer: *So if the kids are playing --*

Participant: *Oh! And they brush their teeth too there. They actually have you, after their snack, they actually have you brush your teeth.*

Interviewer: *Is it a group activity?*

Participant: *Yeah. It’s like the home – I think it’s all of the home visitors and all of their childrens* [sic]*.* – Mother of 1 year old child

### Social environment

Some mothers who mentioned brushing occurring in EHS playgroups indicated that their children were still not willing to have their teeth brushed or cleaned, despite observing other children having their teeth brushed. In a context where young children are socializing, such as in the playgroup, there is the opportunity to observe norms among peers.

Participant: *Since we started going to the playgroup, even when she* [child name] *looked at other kids doing it, she wouldn’t. They gave us gauze to clean her teeth, but she wouldn’t let me. She didn’t want to and she didn’t want to.*

Interviewer: *Not even at the group?*

Participant: *No, not even at the group. I used to tell her, “Look at the children. They are all brushing their teeth.” No. She would just look at them like, “What’s wrong with this?” And she gets upset. –* Mother of less than 2 year old child

### Health care system characteristics

Most mothers discussed their own experiences with the dental care system rather than their child’s experiences with a dentist when access to dental care was mentioned. However, as noted earlier, many mothers were highly knowledgeable about oral health, and several indicated learning from healthcare providers (most frequently pediatricians and dentists). There were examples shared of how the health care and dental care system influenced children’s oral hygiene practices.

*Because what they* [dental staff] *have explained to us at the dentist is that if you have cavities, you can pass those cavities to the other children.* – Mother of almost 2 year old

## Discussion

This study contributes to the literature through the qualitative exploration of multiple levels of influence on young children’s oral hygiene practices. Mothers’ identified several child-, family- and community-level influences, which shed light on both potential positive and negative influences on the target behavior. Study results provide insight into the motivations behind child oral hygiene and directions for interventions that EHS HVs could facilitate.

The child’s developmental stage was perceived as a barrier to brushing, though this was not universally expressed in this sample. Some (mostly older) children were compliant and mothers had no challenges with brushing their children’s teeth daily. The normal hygiene routines for nearly all study children appeared optimal overall, with twice-daily brushing reported as happening most of the time. However, these perceptions could be affected by social desirability biases. Children are unique individuals, often with distinct personality characteristics already formed at young ages. While there is mixed evidence about whether or not different child temperaments may be associated with ECC or ECC risk factors [36, 37], child age and temperament may be an important child-level characteristic worth further exploration. Child temperament was not examined in this study, but could have contributed to mothers’ perceptions about what is normal behavior for their child. Child age is related to developmental stage, and young children do also become more independent over time.

Most factors influencing child oral hygiene routines operated at the family-level, predominantly driven by mothers [38]. Many families in two caregiver households split brushing responsibilities, but narratives about the father’s role and influence varied. In several instances, fathers were identified as generally less involved in their child’s oral hygiene routines, and often less supportive of maintaining the home routine. Similar findings have been documented in Latino families with young children in other studies [14, 39]. When both parents do not uphold and reinforce the child’s routine, it has a negative influence on the child’s hygiene behavior [13]. Our results also suggested that young children learned from their mothers and older siblings who modeled and helped with brushing, suggesting that other family members can influence hygiene routines.

Fathers’ involvement in their child’s brushing routines may mitigate child resistance through both parents working together to gain the child’s cooperation, and the child getting consistent messages about oral hygiene and health as a priority from their parents. Several narratives discussed different parenting styles and approaches to hygiene between mothers and fathers. In this study, information was not available about overall family functioning, though family structure was available. Poorer family functioning (for example, poor communication is one dimension of poorer family functioning) and certain parenting styles (authoritarian and permissive) have been associated with worse oral hygiene habits and higher caries experience in young children [40–43]. HVs are uniquely positioned to work closely with families, learn about parenting styles, families’ home routines, and what may work better for each family. Home visitors that can elicit higher parental engagement during visits has been positively associated with better outcomes in other EHS home-based programs [44], although information was not available in this study about how much the EHS HV specifically interacted with or provided oral health information to families.

EHS HV have high potential to become more effective agents for promoting oral health efforts with their families. While oral health is part of the EHS HV curriculum, there are some reports that oral health activities are not always optimally implemented or attended by families [30, 45]. In a Wisconsin EHS program, additional brief oral health training for HVs that included motivational interviewing (MI) and role playing techniques improved their knowledge and efficacy in this area [33]. However, MI has shown mixed results in preventing caries in young children [7] and adolescents [46].

Other important elements for motivating and supporting behavior change include knowledge, skill, and strategies for overcoming challenges. Mothers in this study were familiar with ECC, understood the risk factors and etiology, and spoke knowledgeably about it, often using correct terminology including “caries,” “bacteria,” and “microbes.” Some mothers did use more common terms like “cavities,” but overall, most were describing the problems and disease process in depth, and identified disease prevention as a motivation for regular hygiene. This contrasts other reports that identify more cosmetic reasons motivating parents to teach and perform oral hygiene with young children [47] and limited ECC knowledge among Latino parents [48, 49]. While parental ECC risk factor knowledge is often assessed, it sometimes excludes mothers’ understanding of the role of bacteria. This suggests that ensuring parental awareness of the ECC disease process is critical knowledge and a salient motivator for good oral hygiene practices. Supportive effects could be further enhanced by ensuring information delivery to the whole family. In an EHS intervention, family-focused interventions were more effective than standard didactic video for anticipatory guidance oral health [50]. EHS HVs are well-positioned to potentially reach the whole family and directly observe facilitating or impeding factors for optimal oral hygiene.

EHS HVs can offer tailored support to parents so they can build the skills and confidence in their ability to brush their children’s teeth correctly until the child is at least 8 years old, which AAPD recommends as the age for independent toothbrushing [10]. While caries prevention was noted to be a primary motivator for frequent hygiene practices, and most mothers reported regularly brushing their children’s teeth, there was a striking knowledge gap, and mothers were not sure when children should be able to brush their own teeth without parental help. This is a notable point for educational intervention that EHS HVs could emphasize. Hoeft and colleagues [51] reported that Latino parents initiated toothbrushing close to age 2 (mean ± standard deviation: 1.8 ± 0.8 years), and believed children could brush independently by about age 3 (3.1 ± 1.2 years). Maternal acculturation level may also play a role in health beliefs and behaviors, and this study sample was predominantly Spanish-speaking mothers. Language is not the only acculturation measure, but there have been differences in the oral health-related knowledge and oral health status of Latino children of Spanish-speaking versus English-speaking mothers, with higher knowledge and better outcomes among children of English-speaking mothers [52]. EHS HV can work with families at home, and reinforce accurate knowledge and oral health-promoting practices. Indeed, results of this study informed a behavioral intervention, which emphasized the mothers’ high knowledge and positive attitudes, by addressing knowledge gaps, reinforcing correct knowledge and attitudes that motivated behavior.

Notably many mothers had effective strategies to engage their children on brushing their teeth regularly—experiences that could be shared with other families through the EHS HV program parent groups and playgroups. Enhancing coping skills of parents who encounter resistance from their young children for brushing could be another intervention opportunity to support parents in successfully brushing young children’s teeth and establish oral hygiene routines and practices. Although mothers in this study did not discuss psychosocial factors like perceived confidence in their ability to brush their child’s teeth well, self-efficacy is a modifiable perception that can be enhanced to improve behavioral routines. Indeed, parents of preschool-aged children with higher brushing efficacy brushed their children’s teeth more frequently [53]. Higher oral health knowledge is also positively associated with self-efficacy and oral health outcomes among young Latino children [54]. Thus, hands-on instruction, demonstrations, practice, and feedback can enhance skills and mothers’ confidence and ability to perform proper oral hygiene for their young children. Building children’s oral hygiene practices into their bedtime routine is recommended as recommended by the American Academy of Pediatrics’ Brush-Book-Bed program [55]. Additionally, other potentially influential family-level psychosocial factors not captured in this study may be important factors in motivating behavior change. For example, in another study of Latino mothers of preschool-aged children, parenting stress was a barrier to children’s oral hygiene [14].

Mothers discussed their own dental care experiences, but were not asked directly about, and did not share much information related to, their young children’s dental utilization. Dental providers possibly could influence parents and provide proper hygiene instruction and support for preschoolers at their first dental visits along with anticipatory guidance [56], yet this potential source of information and influence did not come up in interviews, perhaps due in part to the questions emphasizing oral hygiene behaviors. This particular EHS program was co-located with a FQHC, so dental services may have been more accessible for study participants. It is noteworthy that a few mothers indicated that dental providers did influence knowledge about ECC, and there is potential for dentists to play a larger role in establishing an ideal oral hygiene routine for young children. The health care system is an important community-level influence for oral health promotion in earliest childhood.

Our study findings also suggest that community-level influences on child behavior matter, and there are unique opportunities within the EHS HV program and ways they bring families together and interact with them. Some mothers noted that young children saw peers get their teeth brushed as part of the normal, expected routine after snacking at the EHS playgroups—an opportunity that parents and HVs can use foster positive peer influences. There may also be opportunities to discuss oral health at parent groups and in relation to other child development milestones, like normal everyday routines, such as washing the children’s hands or changing their diapers, or capitalize in other ways on the EHS HV program structure. Thus, EHS HVs can potentially further amplify the effects of their oral health education at the playgroups and family activities. In general, relatively little research has specifically explored the community-level influences on young children’s oral health. Other qualitative studies of young children’s parents show that those without external social support were less likely to brush their preschool-aged children’s teeth twice daily [57] and that parents’ perceptions about child brushing norms were defined relative to how often other parents brushed their children’s teeth [47]. Social influences on children’s hygiene behavior should be studied further.

In sum, child-, family- and community-level factors are important to consider to inform developing tailored interventions and relevant oral health preventive care programs for families in EHS HV programs. The conceptual framework by Fisher-Owens and colleagues [4] was a useful tool for organizing and examining complex and interconnected levels of influence on child oral hygiene behavior, and identifying specific directions for oral hygiene promotion efforts. Several positive influences and opportunities for intervention were noted. Mothers’ high oral-health related knowledge and motivation to brush their child’s teeth were emphasized, and these results informed targeting regular morning and evening toothbrushing.

This study’s strengths and limitations should be considered when interpreting results. This study was conducted with a convenience sample of predominantly foreign-born, Spanish-speaking mothers recruited from one EHS site, which may affect generalizability. Recruitment and scheduling continued until the target sample size (*N* = 25) was enrolled, which could have resulted in some respondent bias. There may have also been some effort justification bias, as participants received an incentive. Many mothers who were initially interested provided minimal availability, predicted that their children would be disruptive during the interview or faced transportation barriers, and opted not to participate. Mothers’ descriptions of their children’s hygiene behaviors could have been subject to social desirability or recall biases. The semi-structured interview approach was appropriate and allowed mothers to openly share their perspectives and explain how different factors affect their child’s oral hygiene practices. However, fathers, other caregivers, and EHS HV perspectives were not captured here, and should be explored in future research. Their perspectives would contribute to a more complete understanding of the EHS HV program context and help inform a future behavioral intervention.

## Conclusion

Multiple factors at child-, family- and community-levels influence young children’s oral health and their oral hygiene practices. EHS HV are well positioned to assess needs and provide support, education, and resources to improve parental toothbrushing techniques and help parents overcome barriers and challenges to promote oral health in earliest childhood.

## Additional file

Additional file 1: Select questions from semi-structured interview guide (Spanish). (DOCX 36 kb)

## Data Availability

The datasets generated and analyzed during the current study are not publicly available due to the data protections for personal identifying information outlined in the IRB protocol for this study, but are available from the study PIs (SG and FRG) on reasonable request.

## Abbreviations

AAPD: American Academy of Pediatric Dentistry
ECC: Early Childhood Caries
EHS: Early Head Start
FQHC: Federally Qualified Health Centers
HV: Home visitor
MI: Motivational interviewing
RA: Research assistant

## References

[1] U. S. Department of Health and Human Services (2000). Oral health in America: a report of the surgeon general - executive summary.

[2] Dye BA, Hsu K-LC, Afful J (2015). Prevalence and measurement of dental caries in young children. Pediatr Dent.

[3] Henshaw MM, Garcia R, Weintraub JA (2018). Oral health disparities across the life span. Dent Clin N Am.

[4] American Academy of Pediatric Dentistry Council on Clinical Affairs, Definition of Early Childhood Caries (ECC). 2008. https://www.aapd.org/assets/1/7/P_ECCClassifications.pdf. Accessed 12 June 2019.

[5] Fisher-Owens SA, Gansky SA, Platt LJ, Weintraub JA, Soobader M-J, Bramlett MD (2007). Influences on children’s oral health: a conceptual model. Pediatrics.

[6] Albino J, Tiwari T (2016). Preventing childhood caries: a review of recent behavioral research. J Dent Res.

[7] Henshaw MM, Borrelli B, Gregorich SE, Heaton B, Tooley EM, Santo W (2018). Randomized trial of motivational interviewing to prevent early childhood caries in public housing. JDR Clin Transl Res.

[8] Franzman MR, Levy SM, Warren JJ, Broffitt B (2004). Tooth-brushing and dentifrice use among children ages 6 to 60 months. Pediatr Dent.

[9] Hooley M, Skouteris H, Boganin C, Satur J, Kilpatrick N (2012). Parental influence and the development of dental caries in children aged 0–6 years: a systematic review of the literature. J Dent.

[10] Leong PM, Gussy MG, Barrow SY, de Silva-Sanigorski A, Waters E (2013). A systematic review of risk factors during first year of life for early childhood caries. Int J Paediatr Dent.

[11] American Academy of Pediatric Dentistry Clinical Affairs Committee (2016). Guideline on infant oral health care, 2014. Ref Manual Clin Pract Guidel.

[12] Hoeft KS, Barker JC, Masterson EE. Maternal beliefs and motivations for first dental visit by low-income Mexican-American children in California. Pediatr Dent. 2011;33(5):392–8. PMC3536823 22104706

[13] Duijster D, de Jong-Lenters M, Verrips E, van Loveren C (2015). Establishing oral health promoting behaviours in children – parents’ views on barriers, facilitators and professional support: a qualitative study. BMC Oral Health.

[14] Finlayson TL, Beltran NY, Becerra K (2017). Psychosocial factors and oral health practices of preschool-aged children: a qualitative study with Hispanic mothers. Ethn Health.

[15] Harris R, Nicoll AD, Adair PM, Pine CM (2004). Risk factors for dental caries in young children: a systematic review of the literature. Comm Dent Health.

[16] Grindefjord M, Dahllof G, Modeer T (1995). Caries development in children from 2.5 to 3.5 years of age: a longitudinal study. Caries Res.

[17] Greenwell A, Johnsen D, DiSantis T, Gerstenmaier J, Limbert N (1990). Longitudinal evaluation of caries patterns from the primary to the mixed dentition. Pediatr Dent.

[18] Castilho ARF, Mialhe FL, Barbosa TS, Puppin-Rontani RM (2013). Influence of family environment on children's oral health: a systematic review. J Pediatr.

[19] Chaudry A, Pedroza JM, Sandstrom H, Danziger A, Grosz M, Scott M (2011). Child care choices of low-income working families.

[20] Early Childhood Learning and Knowledge Center. Washington DC. US. Department of Health and Human Services. https://eclkc.ohs.acf.hhs.gov/. Accessed 22 Feb 2019.

[21] Head Start Program Performance Standards https://eclkc.ohs.acf.hhs.gov/sites/default/files/pdf/ehs-infant-toddler-hspps-chart.pdf. Accessed 22 Feb 2019.

[22] Kranz AM, Rozier RG (2011). Oral health content of early education and child care regulations and standards. J Public Health Dent.

[23] US Department of Health and Human Services, Administration for Children and Families, Early Head Start Programs. Program Information Report, Head Start. https://eclkc.ohs.acf.hhs.gov/sites/default/files/pdf/2017-2018-hs-pir-form.pdf. Accessed 22 Feb 2019.

[24] American Academy of Pediatric Dentistry Council on Clinical Affairs, Definition of Dental Home. 2018. https://www.aapd.org/media/Policies_Guidelines/D_DentalHome.pdf. Accessed 12 June 2019.

[25] US Department of Health and Human Services, Administration for Children and Families, Early Head Start Programs. https://eclkc.ohs.acf.hhs.gov/programs/article/early-head-start-programs. Accessed 22 Feb 2019.

[26] National Resource Center for Health and Safety in Child Care and Early Education. Caring for our children. 3.1.5.3: Oral Health Education. http://nrckids.org/CFOC/Database/3.1.5.3. Accessed 22 Feb 2019.

[27] National Resource Center for Health and Safety in Child Care and Early Education, 3.1.5.1: Routine oral hygiene routines. http://nrckids.org/CFOC/Database/3.1.5.1*.* Accessed 22 Feb 2019.

[28] National Resource Center for Health and Safety in Child Care and Early Education. 3.1.5.2: Toothbrushes and toothpaste. http://nrckids.org/CFOC/Database/3.1.5.2. Accessed 22 Feb 2019.

[29] Kranz AM, Rozier RG, Zeldin LP, Preisser JS (2012). Oral health activities of early head start and migrant and seasonal head start programs. J Health Care Poor Underserved.

[30] Kranz AM, Rozier RG, Zeldin LP, Preisser JS (2011). Oral health activities of early head start teachers directed toward children and parents. J Public Health Dent.

[31] Mofidi M, Zeldin LP, Rozier RG (2009). Oral health of early head start children: a qualitative study of staff, parents, and pregnant women. Am J Public Health.

[32] Martin LT, Karoly LA. Addressing oral health in head start: insights from the head start health manager descriptive study, OPRE report 2016–84. Washington, DC: Administration on Children and Families, US Department of Health and Human Services; 2016.

[33] Glatt K, Okunseri C, Flanagan D, Simpson P, Cao Y, Willis E (2016). Evaluation of an oral health education session for early head start home visitors. J Public Health Dent.

[34] Mariani M, Velazquez L, Kattlove J. Healthy mouth, healthy start: Improving oral health for young children and families through early childhood home visiting. Los Angeles: The Children's Partnership; 2016. https://childrenspartnership.org/wp-content/uploads/2019/05/Healthy-Mouth-Healthy-Start-Improving-Oral-Health-for-Young-Children-and-Families-Through-Early-Childhood-Home-Visiting_August-2016.pdf. Accessed 24 July 2019.

[35] Auger A, Karoly LA, Martin LT. Family engagement in the delivery of the health services component in Head Start and Early Head Start, OPRE report 2016–86. Washington, DC: Administration on Children and Families, US Department of Health and Human Services; 2016.

[36] Quinonez RB, Keels MA, Vann WF, McIver FT, Heller K, Whitt JK (2001). Early childhood caries: analysis of psychosocial and biological factors in a high-risk population. Caries Res.

[37] Spitz AS, Weber-Gasparoni K, Kanellis MJ, Qian F (2006). Child temperament and risk factors for early childhood caries. J Dent Child.

[38] Detwiler DJ (1981). Mother knows best: brushing baby teeth. Dent Stud.

[39] Swan MA, Barker JC, Hoeft KS (2010). Rural Latino farmworker fathers’ understanding of children's oral hygiene practices. Pediatr Dent.

[40] Duijster D, Verrips GHW, Loveren C (2013). The role of family functioning in childhood dental caries. Community Dent Oral Epidemiol.

[41] Duijster D, O'Malley L, Elison S, Van Loveren C, Marcenes W, Adair PM (2013). Family relationships as an explanatory variable in childhood dental caries: a systematic review of measures. Caries Res.

[42] Howenstein J, Kumar A, Casamassimo PS, McTigue D, Coury D, Yin H (2015). Correlating parenting styles with child behavior and caries. Pediatr Dent.

[43] Kitsaras G, Goodwin M, Allan J, Kelly MP, Pretty IA (2018). Bedtime routines child wellbeing and development. BMC Public Health.

[44] Shanti C (2017). Engaging parents in early head start home-based programs: how do home visitors do this?. J Evid Inf Soc Work.

[45] Mathu-Muju K, Lee JY, Zeldin LP, Rozier RG (2008). Opinions of early head start staff about the provision of preventive dental services by primary medical care providers. J Public Health Dent.

[46] Wu L, Gao X, Lo ECM, Ho SMY, McGrath C, Wong MCM (2017). Motivational interviewing to promote oral health in adolescents. J Adolesc Health.

[47] Trubey RJ, Moore SC, Chestnutt IG (2013). Parents’ reasons for brushing or not brushing their child's teeth: a qualitative study. Int J Paediatr Dent.

[48] Tiwari T, Rai N, Colmenero E, Gonzalez H, Castro M (2017). A community-based participatory research approach to understand urban Latino parent’s oral health knowledge and beliefs. Int J Dent.

[49] Hoeft KS, Barker JC, Masterson EE (2010). Urban Mexican-American mothers’ beliefs about caries etiology in children. Community Dent Oral Epidemiol.

[50] Wilson LB, DeBaryshe B, Singh M, Taba S (2013). Evaluating two oral health video interventions with Early Head Start families. Int J Dent.

[51] Hoeft KS, Masterson EE, Barker JC (2009). Mexican American mothers’ initiation and understanding of home oral hygiene for young children. Pediatr Dent.

[52] Tiwari T, Mulvahill M, Wilson A, Rai N, Albino J (2018). Association between maternal acculturation and health beliefs related to oral health of Latino children. BMC Oral Health.

[53] Finlayson TL, Siefert K, Ismail AI, Sohn W (2007). Maternal self-efficacy and 1-5 year old children’s brushing habits. Community Dent Oral Epidemiol.

[54] Wilson AR, Mulvahill MJ, Tiwari T (2017). The impact of maternal self-efficacy and oral health beliefs on Early Childhood Caries in Latino children. Front Public Health.

[55] American Academy of Pediatrics. Brush, Book, Bed. https://www.aap.org/en-us/advocacy-and-policy/aap-health-initiatives/Oral-Health/Pages/Brush-Book-Bed.aspx. Accessed 22 Feb 2019.

[56] Ramos-Gomez F, Crall JJ, Gansky SA, Slayton RL, Featherstone JDB (2007). Caries risk assessment appropriate for the age 1 visit (infants and toddlers). CDA J.

[57] Huebner CE, Riedy CA (2010). Behavioral determinants of brushing young children’s teeth: implications for anticipatory guidance. Pediatr Dent.

