# Supplementary material for: A qualitative study of the multi-level influences on oral hygiene practices for young children in an Early Head Start program
Source: BMC Oral Health. 2019 Jul 26;19:166. doi: 10.1186/s12903-019-0857-7 (PMC6660967; doi:10.1186/s12903-019-0857-7)
Supplement: Supplementary file 1 — Select questions from semi-structured interview guide (Spanish). (DOCX 36 kb) [file 12903_2019_857_MOESM1_ESM.docx]

**Table S1. Select questions from semi-structured interview guide (Spanish)**

1. ¿Puede por favor contarme acerca de su familia o de la gente con la que usted vive?

*Si el encuestado tiene problemas puede ayudar a iniciar con las siguientes preguntas:*

- ¿Con quién vive?
- ¿Cuántos niños tiene?
- ¿Cuántos años tiene su niño que participa en el programa de EHS?

1. ¿Qué tanto conoce a otros padres y niños en el programa de Early Head Start?

- ¿Qué tan a menudo los ve y/o habla con ellos?
- ¿Qué tipo de cosas habla con ellos?

1. Como usted sabe nuestra investigación está tratando de aprender más acerca de la salud oral. ¿Puede contarme que hace regularmente para mantener **su propira** salud oral?

- ¿Cuándo prefiere usualmente cepillarse? ¿Por qué?
- ¿Qué tan parecido es esto para el resto de adultos en su familia?
- ¿Realizaría algún cambio para mantener su salud oral?

1. Nuestro estudio se enfoca en niños de menos de 4 años. ¿Cómo mantienen saludables usted o su familia los dientes de su niño (s) de menos de 4 años?
   - ¿A qué hora del día ocurre normalmente el cepillado (mañana, noche, ambos, en otras horas)?
   - ¿Por qué son esas las mejores horas para cepillarse?
   - ¿Por qué se cepillan con esa frecuencia?
2. ¿Cómo aprendió su niño a cepillar sus dientes?
   - ¿A qué edad comenzó usted cepillar los dientes de su niño? ¿Por qué?
   - ¿A qué edad espera que un niño comience a cepillar sus propios dientes? ¿Por qué?
3. ¿Cuáles son, en su opinión, los aspectos más importantes para el cuidado de los dientes de su niño? ¿Por qué?
   - ¿Cuáles son sus mayores preocupaciones?
   - ¿Qué tan probable es que su niño tenga inconveniencias/malestares con sus dientes? ¿Por qué?
4. **[Si el participante tiene niños más grandes]** Usted mencionó que tiene niños mayores. ¿Hubo experiencias que tuvo con sus hábitos de cepillado de dientes que hayan influenciado la manera como lo hace ahora con su niño más pequeño? Si es así, ¿Qué puede decirme al respecto?
5. Para ayudarme a entender más acerca de la vida de los padres, me gustaría conocer más acerca de su rutina diaria. Por favor piense en esta mañana. ¿Podría describirme que hizo para alistarse para el día?

- Cuénteme acerca de los niños. ¿Quién alistó a los niños pequeños y cómo le fue con eso?
- ¿En qué momento se incluyó el cepillado de dientes en su rutina matutina diaria?
  - ¿Quién se hace cargo del cepillado de su niño(s)?
- ¿El cepillado sí se realizó según lo planeado? ¿Qué sucedió?
  - ¿Qué tan fácil o difícil fue completar el cepillado?
  - ¿Qué hizo para mantener a su niño interesado mientras le cepillaba los dientes? (p.e., ¿Realizó alguna otra actividad mientras se cepillaban?) ¿Qué tan bien funcionó eso?
  - ¿Qué tanto tiempo toma todo el proceso?
- ¿Esto es lo que normalmente sucede en las mañanas? ¿Por qué o por qué no?
  - ¿Qué tan similar o diferente son los fines de semana respecto a los otros días?

1. De manera similar, quiero tener una idea general de lo que sucede en las noches. Por favor piense en lo que hizo anoche antes de acostarse. ¿Podría describirme los pasos que seguía mientras se alistaba para ir a la cama?

- Cuénteme acerca de los niños. ¿Quién alistó a los niños pequeños para ir a la cama y cómo le fue con eso?
- ¿En qué momento se incluye el cepillado de dientes en su rutina nocturna?
  - ¿Quién se hace cargo del cepillado de su niño(s)?
- ¿El cepillado sí se realizó según lo planeado? ¿Qué sucedió?
  - ¿Qué tan fácil o difícil fue completar el cepillado?
  - ¿Qué hizo para mantener a su niño interesado mientras le cepillaba los dientes? (p.e., ¿Realizó alguna otra actividad mientras se cepillaban?) ¿Qué tan bien funcionó eso?
  - ¿Qué tanto tiempo toma todo el proceso?
- ¿Esto es lo que normalmente sucede en las noches? ¿Por qué o por qué no?
  - ¿Qué tan similar o diferente son los fines de semana respecto a los otros días?

1. ¿Qué es lo que usualmente impide que lave los dientes de su niño?
2. ¿De qué maneras puede usted sobrepasar estas dificultades?
